# Supplementary material for: A novel peptidoglycan deacetylase modulates daughter cell separation in E. coli
Source: bioRxiv. 2025 Feb 19:2025.02.18.638797. Preprint. [Version 1] doi: 10.1101/2025.02.18.638797 (PMC11870482; doi:10.1101/2025.02.18.638797)
Supplement: Supplement 1 [file NIHPP2025.02.18.638797v1-supplement-1.pdf]

bioRxiv preprint doi: https://doi.org/10.1101/2025.02.18.638797; this version posted February 19, 2025. The copyright holder for this preprint (which was not certified by peer review) is the author/funder, who has granted bioRxiv a license to display the preprint in perpetuity. It is made available under aCC-BY 4.0 International license.

SUPPLEMENTARY INFORMATION CAPTIONS

S1 Fig. Verification by MS/MS of the identity of peaks for Glc*N*Ac-Mur*N*AcAnh and Glc*N*Ac-Mur*N*AcAnh in the MltA-digested PG samples. (A, B) Chemical structures of Glc*N*Ac-Mur*N*AcAnh and Glc*N*-Mur*N*AcAnh, respectively. **(C)** Verification using MS/MS (bottom) that the peak with m/z 437 (top) corresponds to Glc*N*-Mur*N*AcAnh. Fragmentation of Glc*N*-Mur*N*AcAnh yields Glc*N*(-H_2O)+H^+ (m/z 162), and not Glc*N*Ac(-H_2O)+H^+ (m/z 204), and Mur*N*AcAnh+H^+ (m/z 276). **(D)** Verification using MS/MS (bottom) that peak with m/z 479 (top) corresponds to Glc*N*Ac-Mur*N*AcAnh. Fragmentation of Glc*N*Ac-Mur*N*AcAnh yields the Glc*N*Ac(-H_2O)+H^+ ion (m/z 204) and MurNAcAnh+H^+ (m/z 276).

S2 Fig. Overexpression of full length SddA in the cytoplasm produces a truncated protein. (A) SDS-PAGE and Western-Blot analysis of soluble (*sol*) and *pellet* fractions from sonicated cell extracts of BL21(DE3) pET28a-HisSddA induced with IPTG. The His-tag in the N-terminus His-SddA was detected using anti-His-tag antibody. **(B)** AlphaFold model of *E. coli* SddA showing the predicted globular (red) and unfolded C-terminal (grey) regions, plus the location of cleavage sites in His-SddA purified from soluble extracts as the one analysed in A. The identified cleavage sites are P258, V262, K263 and L264. **(C)** Representative 4-nitrophenyl acetate (4NA) esterase assays with His-SddA. Reactions contained 10 mM 4NA in the presence or absence of 8.8 µM His-SddA (red and black lines, respectively) and were incubated at room temperature.

S3 Fig. SddA is unable to digest peptidoglycan or muropeptides. PG was incubated first with SddA or buffer and then with the muramidase cellosyl (chromatograms labelled "PG") or first with cellosyl and then with SddA or buffer (chromatograms labelled "muropeptides"), to test the activity of SddA on PG or muropeptides, respectively. SddA was added at 10 µM in both cases. SddA treatment did not introduce any changes in the resulting chromatograms.

S4 Fig. SddA can modify denuded glycan strands. (A) Radiolabelled denuded glycan strands were prepared from radiolabelled lipid II ([^{14}C]-lipid II) using transpeptidase-defective PBP1B (S510A), and the amidase AmiC plus its activator NlpD. **(B)** Scheme depicting the preparation of samples (left side) and their analysis (chromatograms on the right side). Muramidase was unable to digest the denuded glycan strands treated with SddA. **(C)** Scheme depicting the preparation of samples (left side) and their

bioRxiv preprint doi: https://doi.org/10.1101/2025.02.18.638797; this version posted February 19, 2025. The copyright holder for this preprint (which was not certified by peer review) is the author/funder, who has granted bioRxiv a license to display the preprint in perpetuity. It is made available under aCC-BY 4.0 International license.

analysis (chromatograms on the right side). SddA modified the short oligosaccharides obtained by digesting radiolabelled denuded glycan strands with a muramidase.

S5 Fig. SddA deacetylates Glc*N*Ac residues in denuded glycan strands. (A) Chromatograms of muramidase-digested denuded glycan strands, treated with SddA (red) or buffer (black) after digestion with the muramidase cellosyl. **(B)** Results of MS and MS/MS analysis of the labelled peaks in A. All muropeptide peaks correspond to the non-reduced species.

S6 Fig. *E. coli* SddA contains conserved residues for Zn^{2+} binding. Sequence alignment of *E. coli* SddA with the sequences of the two proteins with a divergent polysaccharide deacetylase domain (PF04748) whose crystal structure is available. These two proteins are BH1492 from *Bacillus halodurans* (PDB 2NLY), which contains a Zn^{2+} in the crystal structure, and ATU2773 from *Agrobacterium tumefaciens* (PDB 2QV5), which does not contain a Zn^{2+} ion in the crystal structure. The sequences are coloured by secondary structure (red indicating alpha helix and blue beta strand). The residues coordinating Zn^{2+} in BH1492 are highlighted in yellow.

S7 Fig. Protein level of ectopically expressed SddA is higher in a Δ*prc* background. (A) Scheme depicting the cloning of *sddA* with a FLAG tag and its native signal peptide (SP) into pVMH19 and pVMH20 plasmids. (**B)** Expression of SddA from pVMH19 or pVMH20 in BW27883 or BW27883 Δ*prc* was monitored by Western blotting using an anti-FLAG antibody. **(C)** Analysis of the cell extracts used for Western blot in **(B)** using SDS-PAGE and Coomassie staining, showing a uniform level of proteins for all samples. In **(B)** and **(C)**, the expression of FLAG-tagged SddA was induced by incubation with 1mM IPTG for 4 h. **(D)** Left: AlphaFold3 prediction showing an interaction between Prc (colored in pink) and the C-terminal region of SddA (colored in cyan); Middle: focus on the active site residues Ser452 and Lys477 of Prc (CPK representation)[48] interacting with the C-terminal residues KFNASAN of SddA; Right: Predicted Aligned Error (PAE), with SddA top-left and Prc bottom-right (blue, orange and white coding for high, medium and low prediction confidence, respectively).

S8 Fig. Deletion of *sddA* does not cause morphological changes when growing in LB. (A) BW25113 or BW25513 Δ*ssdA*::kan were grown in LB at 37°C. Samples were collected (arrows), fixed, stained with

bioRxiv preprint doi: https://doi.org/10.1101/2025.02.18.638797; this version posted February 19, 2025. The copyright holder for this preprint (which was not certified by peer review) is the author/funder, who has granted bioRxiv a license to display the preprint in perpetuity. It is made available under aCC-BY 4.0 International license.

FM5-95 (cell membrane), immobilized and imaged by phase contrast and epifluorescence microscopy. Representative images are shown. Scale bar is 10 µm. **(B)** morphological measurements and growth doubling time of the cells and growth curves shown in A.

S9 Fig. Deletion of *actS*, *nlpD* or *envC* does not affect OM permeability of cells lacking SddA. (A) Overnight cultures of BW25113 and isogenic Δ*sddA,* Δ*envC,* Δ*actS,* Δ*envC* Δ*sddA,* Δ*actS* Δ*sddA* mutants and **(B)** of BW25113 Δ*nlpD* and isogenic Δ*nlpD* Δ*sddA* were serially diluted and spotted on LB with 5% NaCl plates containing vancomycin (Van), rifampicin (Rif), novobiocin (Nov), mecillinam (Mec), cefsulodin (Cefs), bacitracin (Bac) or SDS/EDTA at the indicated concentrations. Plates were incubated at 37°C for 24 h.

S10 Fig. SddA deletion increases the sick phenotype of Δ*envC* Δ*nlpD*. BW25113 Δ*envC* Δ*nlpD* and BW25113 Δ*envC* Δ*nlpD* Δ*sddA* were grown in LB at 37°C. At indicated times, samples were taken and imaged on agarose pads containing FM5-95 membrane stain. All scale bars represent 10 µM.

S11 Fig. Co-expression of SddA and NlpD does not rescue OM defects and cell chaining. (A) Overnight cultures of BW25113 harbouring empty pGS100 or pBAD24 plasmids (-/-) or pGS100 with *sddA* (*sddA*/-) or pBAD24 with *nlpD* (-/*nlpD*) or both (*ssdA*/*nlpD*) were serially diluted and spotted onto LB-Lennox supplemented with 25 µg ml^{-1} chloramphenicol and 100 µg ml^{-1} ampicillin containing vancomycin (Van), bacitracin (Bac) or SDS/EDTA at the indicated concentrations. 0.2% arabinose and 0.5 mM IPTG were used to induce *nlpD* and *sddA* expression, respectively. **(B)** BW25113 cells harbouring pGS100 and pBAD24 or pBAD24 with *nlpD* (pGS100/*nlpD*), pGS100 with sddA (*sddA*/pBAD24) or both (*ssdA*/*nlpD*) were grown in LB with 5% NaCl supplemented with 25 µg ml^{-1} chloramphenicol and 100 µg ml^{-1} ampicillin. IPTG (0.5 mM) and arabinose (0.2%) were used to induce the expression of *sddA* and *nlpD*, respectively. Samples were collected at the exponential growth phase (arrows) stained with FM5-95 (red, cell membrane) and DAPI (blue, nucleoid), immobilized and imaged by confocal fluorescence microscopy. Representative images are shown. Scale bar is 5 µm.

S12 Fig. Cell chaining phenotype caused by SddA overproduction is independent of EnvC or FtsX. (A) BW25113 (WT) and BW25113 Δ*envC* (Δ*envC*) cells harbouring pGS100 (ev) or pGS100 expressing *sddA*

bioRxiv preprint doi: https://doi.org/10.1101/2025.02.18.638797; this version posted February 19, 2025. The copyright holder for this preprint (which was not certified by peer review) is the author/funder, who has granted bioRxiv a license to display the preprint in perpetuity. It is made available under aCC-BY 4.0 International license.

(SddA) were grown at 37°C in LB with 20 µg ml^{-1} chloramphenicol and expression was induced with 0.5 mM IPTG for 140 min. Samples were imaged by phase contrast and fluorescence microscopy (FM5-95). **(B)** BW25113 (WT) and BW25113 Δ*ftsX* (Δ*ftsX*) cells harbouring pGS100 (ev) or pGS100 expressing *sddA* (SddA) were grown at 30°C in LB with 20 µg ml^{-1} chloramphenicol supplemented with 0.2 M sucrose and expression was induced with 0.5 mM IPTG for 230 min. Samples were imaged by phase contrast and fluorescence microscopy (FM5-95). Representative images are shown. Scale bar is 5 µm.

S13 Fig. High *sddA* expression in mutants defective in amidase activation aggravates their cell chaining phenotype. **(A)** Δ*amiC* Δ*nlpD* and Δ*amiB* Δ*amiA* Δ*envC* cells harbouring pGS100 or pGS100 expressing *sddA* were grown in LB with 5% NaCl supplemented with chloramphenicol at 25 µg ml^{-1}. Samples were collected (arrows) stained with FM5-95 (red, cell membrane) and DAPI (blue, nucleoid), immobilized and imaged by confocal fluorescence microscopy. Representative images are shown. Scale bar is 5 µm. **(B)** Doubling time of cultures shown in panel A.

S14 Fig. Stability of overproduced SddA-sfGFP constructs. BW25113 cells harbouring pGS100 (ev) or pGS100 expressing *sddA*, *sddA::sfgfp* (WT) or *sddA D179A::sfgfp* (D179A) were grown in LB with 20 µg ml^{-1} chloramphenicol at 37°C and expression of the fluorescent constructs was induced with 0.5 mM IPTG for 140 min. Samples were pelleted, protein extracts were obtained by sonication and quantified by BCA. 15 µg of each extract was loaded per lane, separated by SDS-PAGE and GFP was immunodetected by Western blot using α-GFP antibody. (*, unspecific bands)

S15 Fig. Sdd*A* localization is dependent on septal peptidoglycan synthesis. BW25113 cells harbouring pGS100 (ev), pGS100 encoding *sddA, sddA D179A, sddA::sfgfp,* or *sddA D179A::sfgfp* were grown in LB with 20 µg ml^{-1} of chloramphenicol at 37°C. When indicated, 1 µg ml^{-1} of aztreonam was added. Expression of the plasmid-encoded constructs was induced 30 min later by addition of 0.5 mM IPTG for 80 min. Samples were imaged by phase contrast and fluorescence microscopy (FM5-95 or GFP). Representative images are shown. Scale bar is 5 µm.

S16 Fig. **Alphafold-predicted interaction between SddA and EnvC or SddA and FtsX.** Best models obtained by Alphafold of hypothetical SddA-EnvC, **(A)**, and SddA-FtsX complexes, **(B)**; and their

bioRxiv preprint doi: https://doi.org/10.1101/2025.02.18.638797; this version posted February 19, 2025. The copyright holder for this preprint (which was not certified by peer review) is the author/funder, who has granted bioRxiv a license to display the preprint in perpetuity. It is made available under aCC-BY 4.0 International license.

corresponding Predicted Aligned Error (PAE) matrices **(C)** and **(D)**, respectively. Each cell in the PAE matrices represents the estimate alignment error (in Å) between pairs of residues across the predicted protein structure, with darker shades indicating lower PAE and higher model confidence.

S17 Fig. SddA D179A is active against denuded strands and its sfGFP-fusion does not localise to mid-cell and does not cause a cell chaining phenotype. (A) Phase contrast and fluorescence microscopy images of BW25113 harbouring pMP116 (pGS100 *sddA D179A::sfgfp*). Scale bar equals 5 µm. **(B)** Demograpth of SddA D179A-sfGFP localization in cells sorted according to their cell length (from 1.43 µm to 6.4 µm, n = 1555). The fluorescence signal does not show an increase of the intensity at midcell. **(C)** Chromatograms of the analysis of denuded glycan strands treated first with buffer, His-SddA$^{24-238}$ or His-SddA$^{24-238}$ D179A, and then with the lytic transglycosylase MltA. MltA fully digested denuded strands producing anhydro sugars. Reactions contained 2 µM of enzyme and were incubated at 37°C for 24 h. Peak labelled Glc*N* corresponds to Glc*N*-Mur*N*AcAnh and peak labelled Glc*N*Ac corresponds to Glc*N*Ac-Mur*N*AcAnh.

S18 Fig. The mutation D179A induces localized and global changes in SddA dynamics, particularly in regions critical for binding to EnvC. Residue-resolved root mean square fluctuations (RMSF) of wild-type (WT, green) and D179A mutant (red) SddA, calculated from molecular dynamics simulations. The blue curve represents the difference in fluctuations (ΔRMSF) between WT and mutant (WT - mutant). Negative ΔRMSF values indicate greater rigidity in the mutant, while positive values reflect increased flexibility. Residues involved in interaction with EnvC, as predicted by AlphaFold, are highlighted in the analysis.

S19 Fig. SddA does not significantly inhibit ActS-AmiC or EnvC-AmiA. (A, B) HPLC-based end-point activity assays for EnvC-AmiA or ActS-AmiC amidase-activator pairs, in the presence or absence of SddA$^{24-238}$. Sacculi from *E. coli* BW25113Δ6LDT were incubated with 2 µM of the indicated amidase and activator, in the presence or absence of 4 µM His-SddA$^{24-238}$ (SddA$^{24-238}$). EnvC(fl) indicates the EnvC construct containing the LytM and coiled-coiled domain whereas EnvC(LytM) indicates the construct with only the LytM domain. Reactions were incubated for 2 h **(A)** or 1 h **(B)** at 37°C.

bioRxiv preprint doi: https://doi.org/10.1101/2025.02.18.638797; this version posted February 19, 2025. The copyright holder for this preprint (which was not certified by peer review) is the author/funder, who has granted bioRxiv a license to display the preprint in perpetuity. It is made available under aCC-BY 4.0 International license.

Representative chromatograms are shown. **(C)** Chromatograms for the control reactions showing no activation of AmiA or AmiC by $SddA^{24\text{-}238}$. Reactions contained 2 µM of each protein and *E. coli* MC1061 PG and were incubated for 2 h at 37°C. **(D)** Quantification of muropeptides peak areas (peaks 4, 5 and 6) in reactions depicted in A and B, normalized against the areas of those peaks in the controls with no enzyme. Values are average +/- variation of 2 reactions. There was a slight activation of EnvC(LytM)-AmiA activity by SddA. **(E)** Identity and structures of the peaks labelled in A-C and Fig 6A. G stands for Glc*N*Ac, M for Mur*N*AcAnh and G' for Glc*N*.

S1 Table. Strains used in this work.

S2 Table. Plasmids used in this work.

S3 Table. Oligonucleotides used in this work.

S1 References. Supplementary references.
